# Textural radiomic features and time-intensity curve data analysis by dynamic contrast-enhanced MRI for early prediction of breast cancer therapy response: preliminary data

**DOI:** 10.1186/s41747-019-0141-2

**Published:** 2020-02-05

**Authors:** Roberta Fusco, Vincenza Granata, Francesca Maio, Mario Sansone, Antonella Petrillo

**Affiliations:** 1 Radiology Division, Istituto Nazionale Tumori – IRCCS - Fondazione G. Pascale, Via Mariano Semmola, Naples, Italy; 2 Radiology Division, Universita’ Degli Stui di Napoli Federico II, Via Pansini, Naples, Italy; 3 Department of Electrical Engineering and Information Technologies (DIETI), University of Naples Federico II, Via Claudio, Naples, Italy

**Keywords:** Breast neoplasms, Magnetic resonance imaging, Neoadjuvant therapy, Radiomics

## Abstract

**Background:**

To investigate the potential of semiquantitative time-intensity curve parameters compared to textural radiomic features on arterial phase images by dynamic contrast-enhanced magnetic resonance imaging (DCE-MRI) for early prediction of breast cancer neoadjuvant therapy response.

**Methods:**

A retrospective study of 45 patients subjected to DCE-MRI by public datasets containing examination performed prior to the start of treatment and after the treatment first cycle (‘QIN Breast DCE-MRI’ and ‘QIN-Breast’) was performed. In total, 11 semiquantitative parameters and 50 texture features were extracted. Non-parametric test, receiver operating characteristic analysis with area under the curve (ROC-AUC), Spearman correlation coefficient, and Kruskal-Wallis test with Bonferroni correction were applied.

**Results:**

Fifteen patients with pathological complete response (pCR) and 30 patients with non-pCR were analysed. Significant differences in median values between pCR patients and non-pCR patients were found for entropy, long-run emphasis, and busyness among the textural features, for maximum signal difference, washout slope, washin slope, and standardised index of shape among the dynamic semiquantitative parameters. The standardised index of shape had the best results with a ROC-AUC of 0.93 to differentiate pCR *versus* non-pCR patients.

**Conclusions:**

The standardised index of shape could become a clinical tool to differentiate, in the early stages of treatment, responding to non-responding patients.

## Key points

- Significant differences between pathological complete response (pCR) and non-pCR patients were found for texture parameters.
- Standardised Index of shape (SIS) showed the highest accuracy to differentiate pCR patients from non-pCR patients.
- SIS could become a clinical tool to differentiate early responders by non-responders.

## Background

Breast cancer is the most common cancer diagnosed in the USA [1]. Neoadjuvant therapy (NAT) has been recommended in locally advanced disease [2, 3] to determine a downstaging for a following resection to increase tumour control likelihood and breast-conserving surgery rate [4]. Pathologic complete response (pCR) after NAT has been found to be related with long-term clinical benefit, such as disease-free and overall survival [5, 6].

Dynamic contrast-enhanced magnetic resonance imaging (DCE-MRI), being a non-invasive imaging method to measure tissue microvascular perfusion and permeability, is used in clinical trials and research settings to assess NAT response [7]. In clinical settings, changes in tumour size are usually used to assess breast cancer response to NAT. However, changes in tumour size often were found to manifest later compared with changes in vascular tumour functions [8]. There is extensive literature showing that semiquantitative [9] or quantitative pharmacokinetic analysis [10] of DCE-MRI data can provide better prediction, also in early phase, of breast cancer pathologic response to NAT than tumour size changes.

Previous studies have investigated functional parameters derived from DCE-MRI to assess neoadjuvant treatment such as the standardised index of shape (SIS) proposed by Petrillo et al. [11–15] as a simple semiquantitative feature capable to predict pathological significant response and pathological complete response (pCR) after chemo-radiation therapy or after short course radiotherapy. Petrillo et al. demonstrated the ability of SIS to predict pRC and pathological significant response after preoperative chemo-radiotherapy in locally advanced rectal cancer [11–15]. Moreover, texture analysis from breast DCE-MRI has been shown to be effective in applications such as automatic lesion segmentation [16, 17] and cancer diagnosis [18, 19].

Here, we conducted a radiomic analysis of statistical texture features extracted by arterial phase of DCE-MRI and semiquantitative dynamic parameters for early prediction of breast cancer response to NAT. We report our preliminary findings on the performance of these two kinds of data.

## Methods

### Dataset characteristics

Two public dataset were used: ‘QIN Breast DCE-MRI’ and ‘QIN-Breast’.

The public dataset ‘QIN Breast DCE-MRI’ from The Cancer Imaging Archive (TCIA) collection [20, 21] is composed of ten patients subjected to DCE-MRI using a Siemens 3-T TIM Trio system with the body coil and a four-channel bilateral phased-array breast coil. Axial bilateral DCE-MRI images with fat saturation and full breast coverage were acquired with a three-dimensional gradient echo-based time-resolved angiography with stochastic trajectories sequence. DCE-MRI acquisition parameters included echo time 2.9 ms and repetition time 6.2 ms; field of view 30–34 cm, in-plane matrix size 320 × 320; and slice thickness 1.4 mm. The total acquisition time was about 10 min for 32–34 image volume sets of 112–120 slices each, with a temporal resolution of 18–20 s. The contrast agent was Gd-HP-DO3A, gadoteridol (Bracco Imaging, Milan, Italy), intravenously injected (0.1 mmol/kg at 2 mL/s) by a programmable power injector timed to commence after acquisition of two baseline image volumes, followed by a 20-mL saline flush. The public data set can be downloaded at https://wiki.cancerimagingarchive.net/display/Public/QIN+Breast+DCE-MRI.

The public dataset ‘QIN-Breast’ from The Cancer Imaging Archive (TCIA) collection [21, 22] is composed of 35 patients subjected to DCE-MRI using a 3-T Philips Achieva system using a dedicated 16-channel bilateral breast coil. Axial bilateral DCE-MRI images with fat saturation and full breast coverage were acquired with a radiofrequency spoiled three-dimensional gradient echo sequence. Acquisition parameters included echo time 7.9 and repetition time 4.6 ms, field of view 22 cm^2^, in-plane matrix size 192 × 192, and slice thickness 5 mm. For the DCE study, each 20-slice set was collected in 16 s at 25 time points for just under 7 min of dynamic scanning. The contrast agent was Gd-DTPA and gadopentetate dimeglumine (Bayer Health Care Pharmaceuticals, Wayne, NJ, USA) was intravenously injected (0.1 mmol/kg at 2 mL/s) by a programmable power injector timed to commence after acquisition of two baseline image volumes, followed by a 20-mL saline flush. The public data set can be downloaded at https://wiki.cancerimagingarchive.net/display/Public/QIN-Breast.

The NAT protocol administered to these patients was left to the discretion of the treating oncologist based on patient factors such as menopausal status and age as well as tumour characteristics, including size, grade, nodal status and receptor status and was reported by Li et al. in [22]. Both these collections of breast DCE-MRI data contain images from two studies to assess NAT response. Images were acquired at two time points: before and after the first cycle of treatment.

### Data analysis

Manual segmentation was performed by an expert breast radiologist (with a 25-year experience) on the post-contrast arterial phase images, drawing manually each slice to obtain the delineating of the whole tumour contours (volume of interest).

### Textural features

We considered 50 textural features, including both first-order features (mean, mode, median, standard deviation [SD], median absolute deviation, range (absolute difference between maximum and minimum values), kurtosis, skewness, and interquartile range) and second-order features. Calculations were performed using the ‘TextureToolbox’ of MATLAB R2007a (MathWorks, Natick, MA, USA) that performs texture analysis from an input by region or volume of interest. In particular, this texture analysis package allows for wavelet band-pass filtering, isotropic resampling, discretisation length corrections and different quantitation tools. A detailed description has been provided by Vallières et al. [23]. The toolbox can be downloaded at https://it.mathworks.com/matlabcentral/fileexchange/51948-radiomics. The definition of significant textural features reported in the “Results” section is provided in Additional file 1.

### Semiquantitative dynamic parameters

A time-intensity curve can be subdivided into three regions. The first one represents the contrast medium time needed to reach the lesion, and the signal intensity is equal to the basal level before contrast agent injection; the second one shows the increase in signal intensity because of contrast medium absorption (washin) according to the tumour biology; the third one mainly represents the backflow of the contrast medium into the plasma (washout). To estimate shape descriptors, a piecewise linear fitting was made and ten semiquantitative dynamic features described in the literature [24–26] were extracted using the approach reported in a previous publication from our group [25], maximum signal difference (MSD), time to peak between washin and washout segments, washin slope (WIS), washout slope (WOS), washin intercept, washout intercept, area under the curve of washin, area under the curve of washout, and area under the curve of washin and washout. The last semiquantitative dynamic feature was the SIS obtained combining linearly the percentage change of MSD and WOS. Therefore, for SIS calculation, the percentage change of MSD [ΔMSD = (MSD1 - MSD2)/MSD1 × 100], and of WOS [ΔWOS = (WOS1 - WOS2)/WOS1 × 100] and their combination as previously described [11] was evaluated. Standardised SIS was given by the following linear combination: 0.7780*ΔMSD + 0.6157*ΔWOS. In order to evaluate the SIS, an OsiriX (Pixmeo SARL, Geneva, Switzerland) plugin has been developed by the authors.

### Reference standard and pathological methods

The reference standard was the pathology from surgical specimen. Fifteen pCR patients and 30 non-pCR patients were included in this retrospective study. The pCR was classified according to Miller-Payne grade: grade 1 for no reduction, grade 2 for minor loss (≤ 30%), grade 3 for loss from 30 to 90%, grade 4 for marked loss (> 90%), and grade 5 for no residual invasive cancer. Patients with grades 1, 2, 3, or 4 were scored as non-pCR.

### Statistical analysis

Median, SD, and range were calculated as representative values of segmented volumes of interest. Percentage change of median values of parameters obtained before and after the first cycle of treatment was calculated. Receiver operating characteristic analysis was used for obtaining the area under the curve (ROC-AUC). Sensitivity, specificity, positive predictive value (PPV), negative predictive value (NPV), and accuracy were obtained considering the optimal cutoff values identified maximising the Youden index.

For two-group comparisons, we used the non-parametric Kruskal-Wallis test for continuous variables. A *p* value < 0.05 was considered as significant for univariate analysis. Bonferroni correction was applied for multiple comparisons.

Calculations were performed using the Statistics and Machine Learning Toolbox of MATLAB R2007a (MathWorks, Natick, USA).

## Results

Table 1 reports the median, SD, and range of the percentage change for the significant features in the differentiation pCR from non-pCR patients. Significant differences in median values between pCR patients and non-pCR patients using the Kruskal-Wallis test were found for entropy, long-run emphasis (LRE), and busyness among the textural features and for MSD, WOS, WIS and SIS among the dynamic parameters.

**Table 1:** Median, standard deviation and range of the percentage change for significant features differentiating patients with pathologic complete response (pCR) from non-pCR patients

		Δ Entropy (%)	Δ LRE (%)	Δ Busyness (%)	Δ MSD (%)	ΔWIS [%]	ΔWOS [%]	SIS (%)
Non-pCR patients	Median	0.28	0.29	23.79	12.49	38.52	-11.43	9.59
	SD	7.30	2.28	75.93	38.94	50.21	95.11	63.80
	Range	30.57	12.60	298.18	234.22	210.05	420.02	320.65
pCR patients	Median	5.03	1.30	38.83	68.89	87.35	143.67	125.17
	SD	11.69	1.84	37.75	45.30	47.37	129.24	251.58
	Range	43.82	7.52	134.56	142.17	155.66	492.36	779.98
Total	Median	2.16	0.52	27.67	17.00	50.54	20.74	27.96
	SD	9.57	2.21	68.69	43.92	51.28	130.15	183.57
	Range	43.82	12.60	307.92	250.40	224.91	641.45	993.42
*p* value*		0.021	0.024	0.023	0.014	0.012	< 0.001	< 0.001

Range represents the absolute difference between maximum and minimum values

*LRE* Long-run emphasis, *MSD* Maximum signal difference, *SD* Standard deviation, *SIS* Standardised index of shape, *WIS* Washin slope, *WOS* Washout slope

*Kruskal-Wallis test

Table 2 reports accuracy for the significant features: entropy (accuracy 71%), LRE (accuracy 71%), busyness (accuracy 76%), MSD (accuracy 78%), WIS (accuracy 78%), WOS (accuracy 82%), and SIS (accuracy 89%). The SIS showed the best performance with a ROC-AUC of 0.93, a sensitivity of 93%, a specificity of 87%, a PPV of 78%, and a NPV of the 96%, using an optimal cutoff value of 56.47% to differentiate pCR from non-pCR patients. The SIS increased the accuracy of 13% respect to the better parameter among texture features, of 11% compared to MSD and WIS and of 7% respect to WOS.

**Table 2:** Diagnostic accuracy for significant features differentiating patients with pathologic complete response (pCR) *versus* non-pCR patients

		*p* value*	ROC-AUC	Sensitivity	Specificity	PPV	NPV	Accuracy	Cutoff
Textural features	Δ Entropy	0.024	0.71	0.67	0.73	0.56	0.81	0.71	3.78
	Δ LRE	0.021	0.71	0.73	0.70	0.55	0.84	0.71	0.57
	Δ Busyness	0.020	0.72	0.67	0.80	0.63	0.83	0.76	34.38
Dynamic features	Δ MSD	0.013	0.74	0.67	0.83	0.67	0.83	0.78	27.74
	Δ WIS	< 0.001	0.73	0.60	0.87	0.69	0.81	0.78	73.62
	Δ WOS	0.012	0.86	0.87	0.80	0.68	0.92	0.82	24.42
	SIS	< 0.001	0.93	0.93	0.87	0.78	0.96	0.89	56.47

*LRE* Long-run emphasis, *MSD* Maximum signal difference, *NPV* Negative predictive value, *PPV* Positive predictive value, *ROC-AUC* Receiver operating characteristic area under the curve, *SIS* Standardised index of shape, *WIS* Washin, *WOS* Washout slope

*Kruskal-Wallis test

**Fig. 1:**
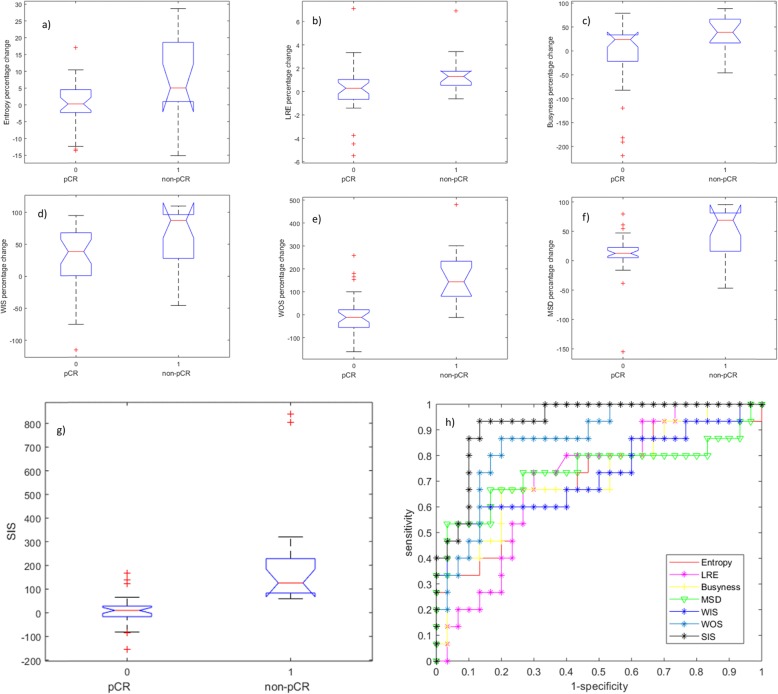
Boxplots for those metrics significantly separating patients with pathologic complete response (pCR) from non-pCR patients. Textural features: (**a**) entropy, (**b**) long-run emphasis (LRE) and (**c**) busyness. Dynamic parameters: (**d**) washin slope (WIS), (**e**) washout slope (WOS), (**f**) maximum signal difference (MSD), (**g**) standardised index of shape (SIS). **h** Receiver operating characteristic area under the curve for all these significant metrics

**Fig. 2:**
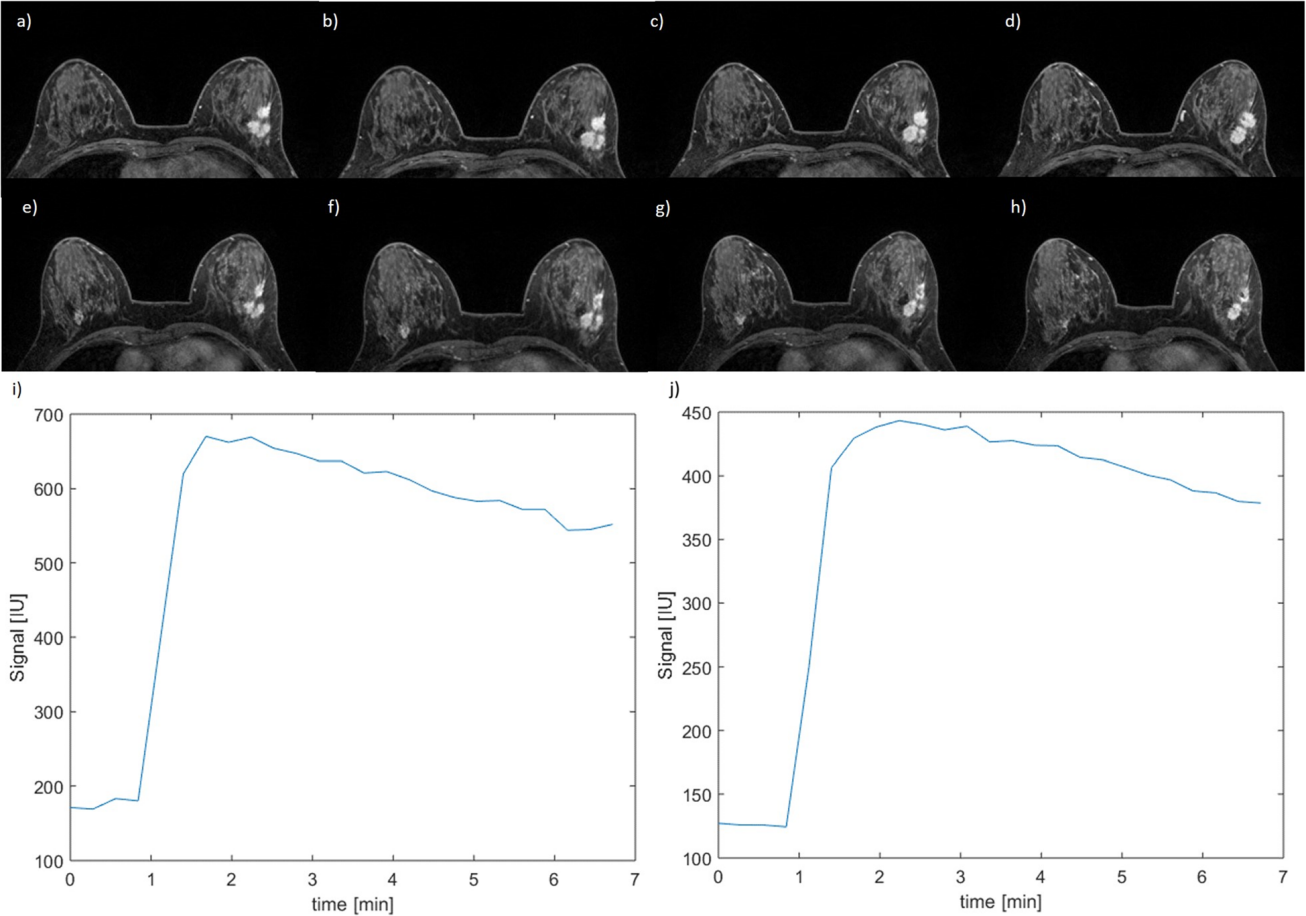
A case of a patient with non-pathologic complete response. **a**–**d** Four slices of T1-weighted dynamic contrast-enhanced magnetic resonance imaging before treatment. **e**–**h** Four slices of T1-weighted dynamic contrast-enhanced magnetic resonance imaging after the first cycle of treatment. **i** Pre-treatment time-intensity curve and (**j**) time-intensity curve after the first cycle of treatment

In Fig. 1, boxplots for the significant textural features (entropy, LRE, busyness) and dynamic features (WIS, WOS, MSD, and SIS) to separate pCR from non-pCR patients are reported. Moreover, Fig. 1 shows ROC-AUC curves for all significant parameters (entropy, LRE, busyness, WIS, WOS, MSD, SIS). In Fig. 2, a case of non-pCR is shown: morphological images did not show a significant change in tumour size while there was a significant modification in time-intensity curve before and after the first cycle of treatment while the SIS value was 72.3%.

## Discussion

Recent advances in biomedical image analysis have emphasised that MRI contrast kinetic parameters and texture analysis, as quantitative metrics, can offer a refined local tumour description of complexity, heterogeneity and kinetic behaviour [27–29].

Teruel et al. [30] presented the findings on 16 textural statistical features extracted by DCE-MRI that are capable to predict early NAT tumour response. Golden et al. [27] used similar texture features to predict pCR, residual lymph node metastases and residual tumour in patients with triple-negative breast cancer. Moreover, Thibault et al. [28] reported that breast tumour microvasculature heterogeneity as a texture feature could be a useful biomarker for early prediction of NAT response. However, these studies used statistical texture description without taking advantage of information provided by the T1-weighted DCE-MRI curve.

Martincich et al. [29] showed that a reduction in the tumour volume > 65% and a reduction in the early enhancement ratio after two cycles of preoperative therapy were associated with a major histopathological response. Combining tumour volume and early enhancement ratio reduction after two cycles of therapy reached a 93% diagnostic accuracy to identify pCR.

We have extracted multiple statistical texture features on arterial phase of DCE-MRI and semi-quantitative kinetic parameters before and after one cycle of NAT in order to assess early pathological response using two public dataset acquired with 3T MR scanner. Our monovariate analysis shows statistically positive results for entropy (71% of accuracy), LRE (71% of accuracy), busyness among texture features (76% of accuracy) and for MSD (78% of accuracy), WIS (78% of accuracy), WOS (82% of accuracy), and SIS (89% of accuracy) among semi-quantitative kinetic metrics. Textural feature results for entropy, LRE and busyness confirmed the results presented by Thibault et al. [28], suggesting changes in the spatial heterogeneity of the tumour microenvironment as one of the initial NAT effects.

Moreover, our perfusion and permeability as semiquantitative dynamic parameters, measured by contrast kinetics, have reported good results, especially WOS and SIS, indicating that changes in DCE-MRI are important markers for identifying early pCR [11, 24, 29].

However, SIS analysis reached the best results in terms of sensitivity, specificity, PPV, and NPV, reporting the highest ROC-AUC value (0.93) for predicting pCR. With the optimal cutoff value, SIS increases the accuracy of 13% compared to the better parameter among texture features, of 11% compared to MSD and WIS and of 7% compared to WOS.

This study has several limitations. First of all, this pool of patients derives from two different public datasets created in two different hospitals with two different MR machines using two different sequence tools. Second, the small cohort of studied patients represents an initial finding to validate increasing sample size of the study in the future. Third, NAT regimen is not available for each patient because the analysed MR images were obtained by public dataset. Finally, this analysis did not consider tumour histological differences. In fact, the potential integration of texture, morphological and dynamic metrics combined with histopathology results may provide other important prognostic information for the assessment and the prediction of therapy response.

In conclusion, although validation in larger patient populations is needed, feature extraction approach and SIS can become important clinical tools to identify and differentiate, in the early stages of NAT treatment, responding and non-responding patients for alternative personalised therapy regimens.

## Supplementary information

**Additional file 1.** Definition of significant texture features

## Data Availability

The datasets used and/or analysed during the current study are available from the corresponding author on reasonable request.

## Abbreviations

DCE-MRI: Dynamic contrast-enhanced magnetic resonance imaging
LRE: Long-run emphasis
MSD: Maximum signal difference
NAT: Neoadjuvant therapy
NPV: Negative predictive value
pCR: Pathological complete response
PPV: Positive predictive value
ROC-AUC: Receiver operating characteristic area under the curve
SIS: Standardised index of shape
WIS: Washin slope
WOS: Washout slope
