# Supplementary material for: Textural radiomic features and time-intensity curve data analysis by dynamic contrast-enhanced MRI for early prediction of breast cancer therapy response: preliminary data
Source: Eur Radiol Exp. 2020 Feb 5;4:8. doi: 10.1186/s41747-019-0141-2 (PMC7002809; doi:10.1186/s41747-019-0141-2)
Supplement: Supplementary file 1 — Additional file 1. Definition of significant texture features [file 41747_2019_141_MOESM1_ESM.docx]

**DEFINITION OF SIGNIFICANT TEXTURE FEATURES**

**Entropy** [1]:


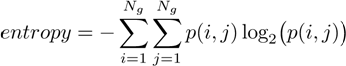


where *P (i, j)* represents the number of times voxels of gray-level *i* were neighbours with voxels of gray-level *j* in the volume *V(x, y, z)*, and *N_g_* represents the pre-defined number of quantized gray-levels set in *V*. The entry *(i,j)* of the of the normalized Gray-Level Co-occurence Matrix (GLCM) is then defined as:


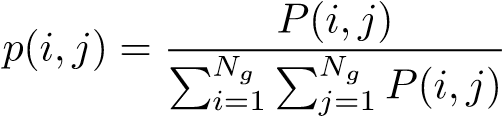


**Long Run Emphasis** (LRE) [2]:


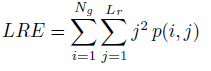


where *P(i,j)* represents the number of runs of gray-level *i* and of length *j* in *V*, *N*g represents the pre-defined number of quantized gray-levels set in *V*, and *L_r_* represents the length of the longest run (of any gray-level) in *V*. The entry (i,j) of the of the normalized Gray-Level Run-Length Matrix (GLRLM) is then defined as:


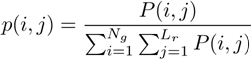


**Busyness** [3]:


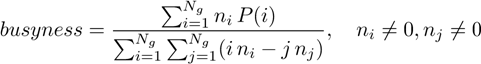


where *P(i)* represents the summation of the gray-level differences between all voxels with gray-level i and the average gray-level of their 26-connected neighbours in 3D space. *N_g_* represents the pre-defined number of quantized gray-levels set in *V*, and *(N_g_)_eff_* is the effective number of gray-levels in *V*, with *(N_g_)_eff_* <*Ng* (let the vector of gray-levels values in *V* be denoted as *g = g(1),g(2),...,g(N_g_)*; some gray-levels excluding *g(1)* and *g(N_g_)* may not appear in V due to different quantization schemes).

The ith entry of the Neighbourhood Gray-Tone Difference Matrix (NGTDM) is then defined as:


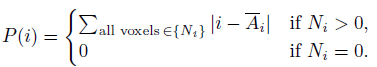


where {Ni} is the set of all voxels with gray-level i in V (including the peripheral region), Ni is the number of voxels with gray-level i in V, and Ai is the average gray-level of the 26-connected neighbours around a center voxel with gray-level i and located at position (j,k,l) in V such that:


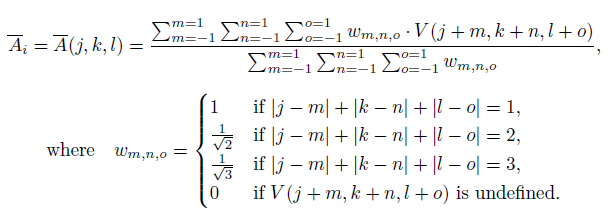


**References**

1. Haralick, R.M., Shanmugam, K. and Dinstein, I. (1973). Textural features for image classification. IEEE Transactions on Systems, Man, and Cybernetics, smc 3(6), 610621.
2. Galloway, M.M. (1975). Texture analysis using gray level run lengths. Computer Graphics and Image Processing, 4(2), 172-179.
3. Amadasun, M. and King, R. (1989). Textural features corresponding to textural properties. IEEE Transactions on Systems, Man, and Cybernetics, 19(5), 1264-1274.
